# Observations on Mental Derangement; Being an Application of the Principles of Phrenology to the Elucidation of the Causes, Symptoms, Nature, and Treatment of Insanity

**Published:** 1831

**Authors:** 


LXIII.

OBSERVATIONS ON MENTAL DERANGEMENT; BEING AN APPLICATION OF THE PRINCIPLES OF PHRENOLOGY TO THE ELUCIDATION OF THE CAUSES, SYMPTOMS, NATURE, AND TREATMENT OF INSANITY. By ANDREW COMBE, M.D. Pp. xxxvi. 392, 12mo. Edinburgh and London, 1831.

DR. COMBE, in these "Observations," exhibits a general view of the brain and nervous system, and shews that, during life, every act of sensation however slight—and every act of perception, however feeble, every affection and moral emotion indeed, and every intellectual operation, are inseparably related *to*, and influenced *by*, a corresponding affection of the material organs with which the Creator has connected them. Hence, he refers the origin of mental derangement to disturbance of cerebral action, and explains how the intensity and proneness to activity of the mind's various faculties, are greatly modified by the relative size of their respective cerebral organs, in conformity with a universal law which pervades animate and inanimate nature, and is applicable to the brain and nervous system in common with all other parts. He maintains, that this condition of relative size, hitherto overlooked, is highly influential in *modifying*, often in *favouring*, the action of the numerous causes of disease by which we are constantly surrounded, and that, next to the hereditary tendency transmitted from parent to child, it is the most powerful of those conditions which predispose an individual to the invasion of insanity. Dr. C. next examines the various conditions required for the healthy action of the brain, and for the full development of the mental powers: in neglect of these conditions, and in contravention of others, he finds fruitful sources of nervous and mental disease: he shews that, in accordance with this observation, every circumstance to which mental derangement can be attributed, acts with a frequency and energy proportioned to the previous susceptibility of the patient, and to the directness with which it affects the nervous function: and, by analyzing the multifarious symptoms accompanying insanity, he discovers most of them in disturbed function of some nervous part, and points out the light in which they ought to be viewed as *signs* of the bodily state from which they originate. He considers next, the duration, periodicity, terminations, and symptomatic forms of the different cerebral affections on which insanity depends, and then explains the affinity existing between these affections and the numerous other disorders to which the nervous system, partially or generally, is liable. Taking a view of the necrotomical appearances connected with deranged mind, the Doctor shews—that the same analogy subsists between the brain and other organs, in its diseases, as holds between them in their healthy conditions;—that, if disorder of cerebral function is sometimes the only *sign* of morbid action, so likewise is disordered function occasionally the only *symptom* of disease in other parts;—that, when mental derangement prevails without leaving, after death, any appreciable change of structure in the brain, or when alterations of the cerebral structure are found where, during life, no sign indicated their presence, so also the exact counterpart of this occurs with other organs in which violent and even fatal disorder takes place, without leaving any organic lesion to mark its previous existence;—and that, on the other hand, organic changes are detected by dissection, in parts where, during life, no corresponding disturbance of function indicated their development. Lastly, combining the results of his previous expositions, Dr. C. sketches out rules of treatment, medical and moral, in harmony with the existing state for which we are required to prescribe, and in harmony also with those leading principles in physiology, therapeutics, and pathology, by which we are accustomed to direct our curative efforts in all other bodily diseases, but which have hitherto been imperfectly applied in treating a class of affections second to none in inherent interest, or in importance to mankind. Dr. C.'s "Observations" abound with the results of deep practical reflection, and must be *studied*, in order to be rightly understood and appreciated: they are distinguished remarkably by the pure eloquence of Candour and the dignified persuasiveness of Philosophy;—altogether, the work is not surpassed by any one, of its kind, in medical science.

Note.—A comprehensive analytical review of Dr. C.'s book is in preparation by one of our correspondents, but the article has been unexpectedly delayed by his other engagements.

